# Editorial: Diabetes and Heart Failure: Pathogenesis and Novel Therapeutic Approaches

**DOI:** 10.3389/fphys.2019.00253

**Published:** 2019-03-19

**Authors:** Claudio de Lucia, Celestino Sardu, Laurent Metzinger, Coert J. Zuurbier

**Affiliations:** ^1^Center for Translational Medicine, Lewis Katz School of Medicine, Temple University, Philadelphia, PA, United States; ^2^Department of Advanced Medical and Surgical Sciences, University of Campania “Luigi Vanvitelli”, Naples, Italy; ^3^EA4666 HEMATIM, CURS, University of Picardie Jules Verne, Amiens, France; ^4^Laboratory of Experimental Intensive Care and Anesthesiology, Anesthesiology, Amsterdam Cardiovascular Sciences, Amsterdam Infection & Immunity, Amsterdam UMC, University of Amsterdam, Amsterdam, Netherlands

**Keywords:** diabetes, heart failure, diastolic dysfunction, arrhythmia, SGLT2 (sodium-glucose cotransporter 2) inhibitor, GLP-1-glucagon-like peptide-1

Type 2 diabetes mellitus (T2DM) and Heart Failure (HF) are two complex multifactorial diseases that can coexist and strongly amplify each other, suggesting overlapping mechanisms contributing to disease state. T2DM patients with HF have a higher risk of mortality and hospitalization for HF than HF patients without T2DM. Therefore, there is an increasing necessity to find new diagnostic instruments and treatments to improve clinical outcomes in T2DM subjects with HF (Maack et al., 2018). Several complex pathological mechanisms such as altered cardiac ionic homeostasis, oxidative stress, hyperglycemia-induced cellular damage, and mitochondrial dysfunction are implicated. Importantly, altered cardiac ionic homeostasis is an established signature and driver of HF pathology (Pogwizd et al., 2003), but has been largely neglected in T2DM pathology. Insulin resistance and disturbances in glucose and fatty acid metabolism are currently viewed as major instigators of T2DM (Jia et al., 2018). However, older and recent researches indicate that cardiac ionic disturbances may actually provide an important common ground for both diseases and explain, at least in part, why they mutually amplify each other. Intriguingly, these changes may lead to electrical and mechanical alterations in systolic and diastolic electrical phases. Therefore, in this Research Topic we have tried to bring the two major diseases together by creating a collection of articles written by authors that have focused on common molecular pathways and mechanisms, electrical/mechanical alterations, and subsequently on clinical outcomes in both T2DM and HF.

In the first review by Borghetti et al. emphasis is placed on focusing of diabetic therapies beyond glucose control. Although anti-hyperglycemic drugs are crucial in the management of diabetes by effectively reducing microvascular complications, preventing renal failure, retinopathy, and nerve damage, they have little effect on diabetic cardiomyopathy. Interestingly, several novel drugs have now shown cardiovascular beneficial effects beyond their ability to control glycemia, such as GLP-1 receptor agonists and sodium-glucose co-transporter 2 inhibitors. In addition, the recent development of modulating the expression of specific cardiac genes or non-coding RNAs *in vivo* for therapeutic purpose, has opened up the possibility to regulate the expression of key players in the development/progression of diabetic cardiomyopathy.

The review by Bajpai and Tilley discusses the roles of leukocytes and particularly neutrophils, macrophages, and lymphocytes in the appearance of myocardial infarction and heart failure during diabetes. Cardiac injury in diabetes, a chronic inflammatory disease, is linked to increased leukocyte mobilization and the expression of pro-inflammatory cytokines and appearance of oxidative stress. The lessons learned from experimental diabetes models in rodents, including the popular streptozotocin-induced Type I diabetes rodent model, are implemented to human patients, and the authors conclude that further studies are necessary to fully apprehend the potential alterations in leukocyte phenotypes and the molecular mechanisms responsible for diabetes.

The review by Grisanti highlights the impact of diabetes on the electrical conduction system in the heart, resulting in atrial fibrillation and ventricular arrhythmias, with a focus on molecular mechanisms, cardiac alterations and therapeutic ameliorations, with a particular emphasis on the contribution of oxidative stress to the pathogenesis of cardiac arrhythmias. The author states that modifications induced by diabetes within the heart change the electrical signaling and conduction in turn altering ion channels and gap junctions' expression and function. Still, antiarrhythmic drugs are effective in the course of diabetes but their mode of action remains to be better characterized.

Uthman et al. dive into direct cardiovascular effects of the novel drug class of SGLT2 inhibitors, the first antidiabetic drugs that were able to reduce hospitalization of heart failure. Although SGLT2 inhibitors were develop to specifically target the kidney (SGLT2 mainly present in kidney), current research demonstrate many additional important and possibly beneficial direct effects of these drugs on endothelial cells and cardiomyocytes. SGLT2 inhibitors can normalize elevated sodium and calcium levels in cardiomyocytes through inhibition of the cardiac sodium-hydrogen exchanger, improve vascular function and activate anti-oxidant systems and intracellular stress signals such as AMPK and reduce inflammatory pathways. Further research will have to demonstrate to what extent these cellular cardiac mechanisms contribute to the large beneficial effects of SGLT2i's in clinical trials. Despa then goes on to describe that sodium is often increased in the failing and diabetic cardiac cell. Elevated intracellular sodium can then cause oxidative stress and augments the sarcoplasmic reticulum Ca^2+^ leak, thus amplifying the risk for arrhythmias and promoting heart dysfunction. Alterations in Na^+^ extrusion and/or Na^+^ uptake that underlie the [Na^+^]_i_ increase in heart failure and diabetes are discussed, and emphasis is placed on the emerging role of Na^+^ -glucose cotransporters in the diabetic heart. Doliba et al. subsequently demonstrate the functional and energetics consequences of elevated sodium in the diabetic cardiomyocyte in their pioneering studies in this field. Their work clearly indicates that raising sodium in the cardiac cell will directly result in impaired mitochondrial function, possibly explaining the often observed energetic problems in the diabetic heart. These reviews all point to the importance of ionic homeostasis and disturbances in the setting of diabetic cardiomyopathy, cardiac arrhythmias, and the elevated incidence of heart failure in T2DM patients.

The article by Liu et al. evaluated the clinical effects of Black garlic in patients with ischemic heart failure. They found that black garlic treatment improved cardiac function in terms of left ventricular ejection fraction and the scores of quality of life (QOL) and decreased circulating BNP precursor N-terminal (Nt-proBNP) by increasing antioxidant levels.

Koyani et al. studied the effects of saxagliptin and sitagliptin (anti-hyperglycemic drugs that have been shown to inhibit dipeptidyl peptidase 4 -DPP4) in mouse ventricular cardiomyocytes. The authors showed that saxagliptin (but not sitagliptin) impaired Ca^2+^ transient relaxation and prolonged action potential duration in cardiomyocytes. They suggested that these results are linked to saxagliptin-DPP9 interaction and following impairment in Ca^2+^/calmodulin- dependent protein kinase II (CaMKII)- phospholamban (PLB) and protein kinase C (PKC) signaling.

Trotta et al. investigated the anti-hypertrophic effect of the melanocortin receptor 5 (MC5R) stimulation in cardiomyocytes exposed to high glucose. The authors showed that MC5R agonists increased viability and reduced total protein in cell stimulated with high glucose via reduced GLUT1/GLUT4 ratio at the plasma membrane and increased intracellular phosphoinositide 3-kinase (PI3K) activity. In addition, MC5R stimulation showed beneficial effects on cardiac function and hypertrophy paralleled by significantly reduced blood glucose levels in a rat model of diabetes (streptozotocin-induced).

We have arrived at exciting times in the world of diabetes and heart failure. At last, several novel antidiabetic drugs have shown large cardiovascular benefits in T2DM patients. Understanding the causal mechanism of these effects are now crucial in order to further our understanding of the interaction between diabetes and heart failure, possibly also offering new therapeutic strategies to combat heart failure in the absence of diabetes.

## Author Contributions

All authors listed have made a substantial, direct and intellectual contribution to the work, and approved it for publication.

### Conflict of Interest Statement

The authors declare that the research was conducted in the absence of any commercial or financial relationships that could be construed as a potential conflict of interest.
